# Determination of Multi-Class Antimicrobial Residues and Antimicrobial Resistance in Cow Milk and Feces Samples during Withdrawal Period

**DOI:** 10.3390/ani13233603

**Published:** 2023-11-22

**Authors:** Zehra Hajrulai-Musliu, Risto Uzunov, Maksud Krluku, Stefan Jovanov, Velimir Stojkovski, Mila Arapcheska, Dea Musliu, James Jacob Sasanya

**Affiliations:** 1 Faculty of Veterinary Medicine, Ss. Cyril and Methodius University, Lazar Pop-Trajkov 5-7, 1000 Skopje, North Macedonia; risteuzunov@fvm.ukim.edu.mk (R.U.); sjovanov@fvm.ukim.edu.mk (S.J.); velimir@fvm.ukim.edu.mk (V.S.); 2 Field Veterinary Practice Maksi, Amdi Leshi 21, 1250 Debar, North Macedonia; maksi_k@yahoo.com; 3 Faculty of Biotechnical Sciences, St. Kliment Ohridski University, Partizanska bb, 7000 Bitola, North Macedonia; mila.arapceska@uklo.edu.mk; 4 Faculty of Pharmacy, Ss. Cyril and Methodius University, Majka Tereza 47, 1000 Skopje, North Macedonia; deamusliu00@hotmail.com; 5 International Atomic Energy Agency, Vienna International Centre, P.O. Box 100, 1400 Vienna, Austria; j.sasanya@iaea.org

**Keywords:** antibiotic residues, antimicrobial resistance, LC-MS/MS, withdrawal period, milk, feces

## Abstract

**Simple Summary:**

Antimicrobial resistance is a worldwide problem that involves humans, animals and environmental health. The extensive use of antibiotics in animal production may be a determining factor for the emergence and dissemination of antibiotic resistance genes throughout the animal production chain. The acquisition of the antibiotic resistance genes in commensal and environmental microorganisms, which usually are susceptible to antimicrobials, can contribute to dissemination of antimicrobial resistance throughout the food chain. This study aimed to determine the levels of residues from five different antimicrobial drugs in milk and feces samples during the withdrawal period in dairy cattle administrated with a single dose of the drug, as well as to characterize the antimicrobial resistance patterns of commensal *Escherichia coli* cultured from feces samples. According to results obtained, the levels of analyzed antimicrobial residues gradually decreased until their elimination (which was on the 7th day after drug administration), and the highest antimicrobial resistance of *E. coli* (100%) was found in β-lactams. The proper drug dosage adjustment and regular monitoring of antibiotics use in animal production is important for in-time identification of antimicrobial resistance in food-producing animals.

**Abstract:**

The use of antimicrobials in livestock production and their effect on the development of antimicrobial resistance (AMR) is a global health problem for humans, animals and the environment. The aim of this study was to determine antimicrobial residue levels in milk and feces samples during the withdrawal period in dairy cattle administrated with a single dose of the drug, as well as to characterize the antimicrobial resistance patterns of *Escherichia coli* cultured from feces samples. In the study, dairy cows from three different farms in North Macedonia were included. Raw milk and feces samples were collected before drug administration (0 day) and on the 1st, 2nd, 3rd, 7th and 21st day after drug administration. The antimicrobial residues of oxytetracycline, enrofloxacin, amoxicillin, trimethoprim and procaine-benzylpenicillin were determined using a validated liquid chromatography combined with tandem mass spectrometry (LC-MS/MS) method involving stable isotopes. According to results obtained, the highest levels of analyzed antimicrobial residues were determined on the first day after drug administration, which then gradually decreased until their elimination (7th day). The highest AMR of *E. coli* (100%) was found in β-lactam antimicrobials. Less exposure to broad-spectrum antimicrobials could be an important factor for reduction of AMR on dairy farms.

## 1. Introduction

Antimicrobials are used in livestock farming for therapeutic purposes, prophylaxis and growth promotion of animals. The inappropriate use of antimicrobials in livestock production contributes significantly to the development of antimicrobial resistance (AMR), which is a growing threat to public health [1]. Many antimicrobials used in humans either belong to the same classes or have the same mechanism of action as those used for animals [2]. Intensive use of antimicrobials in human and veterinary medicine worldwide has accelerated the selection of antimicrobial resistant bacteria [3].

Maximum residue limits (MRLs) for veterinary drugs in foodstuffs of animal origin are set by the decision of the European Commission in the Regulation EU 37/2010 [4]. However, little attention is often paid to the implication of trace residue levels below the MRL, although a direct relationship between antimicrobial use and subsequent AMR has been described [5,6]. Antimicrobial use does not only affect the target pathogen but also the commensal bacteria. Therefore, the gut microbiota is considered as a potential reservoir of resistance genes [7,8]. About 30–90% of the antimicrobials administered for veterinary use can be excreted through feces or urine, either unchanged or as an active metabolite [9,10].

After application, depending on the substance, antimicrobials may be adsorbed and partially metabolized before they are excreted. Very high concentrations varying from 91 mg kg^−1^ to 136 mg kg^−1^ excreted in manure have been reported for antimicrobials such as sulfonamides [11] and tetracyclines [12]. Antimicrobial residues enter the environment either directly by spreading of manure or after collection and storage in the form of sludge. Applied to farmlands, the active ingredients reach the upper soil layer, where they either accumulate or may be rinsed off into surface waters or may leach to groundwater where they can impact both human and environmental health. Massive discharges of antimicrobials into the environment have been reported with high levels in soil [13], sediment [14] as well as surface and groundwater [15]. Such presence is one of the factors contributing to the spread of antibiotic resistance [16].

One of the ways for entrance of antimicrobial residues and antimicrobial resistant bacteria into the environment is application of raw manure on farmlands. The exchange of antimicrobial resistance genes in manure- and soil-associated bacteria can be accomplished by horizontal gene transfer [17]. In soil, antimicrobial residues and resistant bacteria can end up in surface water, through drainage they can contaminate groundwater, and they can also be taken up by crops, thus they present a risk to human and animal health and a risk for the further spread of antimicrobial resistance [18,19,20].

Despite the fact that antimicrobial resistance has a large impact on public health and it is an urgent problem, there is little knowledge about the presence of antimicrobial agents and antimicrobial resistant bacteria in animal manure. The most widely used antimicrobials in cattle production systems are tetracyclines and ionophores. We undertook this research with the following goals: (1) detection and quantification of antimicrobial residues in milk and feces of treated cows; (2) detection and isolation of extended spectrum β-lactamase- (ESBL), AmpC- and carbapenemase-producing *Escherichia coli* from feces samples of the dairy cows; (3) determination of the minimal inhibitory concentration (MIC) of antimicrobials in feces by broth microdilution; (4) determination of the phenotypic profiles of the isolates using commercial microtiter panels.

## 2. Materials and Methods

### 2.1. Animals and Treatment

This study was performed in spring 2021 involving three farms located in western part of North Macedonia. Twenty-five dairy cattle at first lactation, age of two years, were included in the study. They were kept in tie stall housing system. The cattle were chosen randomly, and they were grouped in experimental (*n* = 20) and control group (*n* = 5). The animals in experimental group were with indications of infectious disease or established diagnosis confirmed by a veterinarian. Due to differences in herd sizes of the three farms included in this study, the number of animals in the experimental group was different than control group.

The experimental group of animals had been treated with a single dose administration of: Geomicin R (oxytetracycline 200 mg/mL) for the treatment of infections of the genito–urinary tract; Pen-strept (200,000 IU/mL; dihydrostreptomycin sulphate 200 mg/mL) for treatment of mastitis; Trimetosulfon 48% (sulfadiazine sodium 400 mg/mL and trimethoprim 80 mg/mL) for infections of the respiratory system; Amoximed LA (amoxicillin), 150 mg/mL for local bacterial infection; and Enrocin-S 10% (enrofloxacine 100 mg/mL) for gastrointestinal infections. Selection of animals for antimicrobial treatment was done according to routine diagnostic cases and was based on indications of infectious disease or established diagnosis. The control group was composed of healthy animals, which were not treated with antimicrobial drugs.

### 2.2. Sample Collection

#### 2.2.1. Sample Collection for Antimicrobial Residue Analysis

To assess the concentrations of oxytetracycline, procaine benzylpenicillin, trimethoprim, amoxicillin and enrofloxacin in milk and feces, the samples were collected manually on 0, 1, 3, 5, 7, 14, and 21 days after drug administration. The sample sets consisted of 25 mL of milk and 25 g feces and were collected using sterile plastic screw-caps and then stored at −20 °C until laboratory analysis. Sample collection was done by a veterinarian. Total number of sample sets was 25 (20 sample sets were collected from animals from experimental group, and 5 sets were from animals from control group).

#### 2.2.2. Feces Samples for Antimicrobial Susceptibility Testing (AST)

For detection and isolation of presumable resistant ESBL, AmpC and carbapenemase *Escherichia coli,* feces samples were collected using sterile medical gloves and sterile cups. The samples were transported to the Laboratory of microbiology of the Food Safety Institute, following guidelines in the ISO/DIS 7218 standard [21] Microbiology of food and feed ((EC) No. 625/2017) [22]. The samples were maintained at 5 ± 3 °C and delivered to the laboratory within 4–6 h. The microbiological examination started on the same day.

### 2.3. Identification of Commensal E. coli

First, 1 g of fecal sample was incubated for 18 to 22 h at 37 ± 1 °C after being mixed 1:10 with Buffered Peptone Water (BPW) (Merck, Darmstadt, Germany). Subsequently, a 10 μL loop containing the enriched sample was cultivated on TBX agar (Merck, Darmstadt, Germany). A typical *E. coli* green colony from the TBX plate was subcultured on Nutrient agar (Oxoid, Hampshire, UK) and incubated for 24 h at 37 °C following a 24-h incubation period at 44 °C. Using biochemical assays such the VI-TEK 2 Compact System (BioMérieux, Craponne, France) and indole and oxidase tests (Oxoid, Hampshire, UK), colonies were employed to confirm the presence of commensal *E. coli* after incubation.

### 2.4. Isolation and Identification of Presumptive ESBL/AmpC and Carbapenemase-Producing Commensal E. coli

Presumptive ESBL-, AmpC- and carbapenemase-producing *Escherichia coli* were detected and isolated (EURL-AMR) according to the EU Reference Laboratory for Antimicrobial Resistance’s protocol [22]. For that purpose, 1 g of fecal sample was incubated for 18 to 22 h at 37 ± 1 °C after being mixed 1:10 with Buffered Peptone Water (BPW). The enriched sample was then looped full (10 μL loop) onto MacConkey agar (Oxoid, UK), supplemented with 1 mg/L cefotaxime by streaking and incubated for 18–22 h at 44 ± 0.5 °C. Presumptive ESBL/AmpC-producing *E. coli* on MacConkey + cefotaxime plates showed up as reddish-purple colonies based on colony morphology and color. Using commercial bi-plate selective chromogenic medium (Chromid Carba Smart; BioMérieux, Craponne, France) and the previously described methodology, carbapenemase-producing commensal *E. coli* were detected.

Using the same procedure as previously described, commercial bi-plate selective chromogenic medium (Chromid Carba Smart; BioMérieux, Craponne, France) was utilized to discover carbapenemase-producing commensal *E. coli* [23].

### 2.5. Antimicrobial Susceptibility Test (AST) Determination by Broth Microdilution

Antimicrobial Susceptibility Testing was successfully conducted by microdilution in broth (ISO 20776-1/2 [24,25]), published by EUCAST (European Committee on Antimicrobial Susceptibility Testing), using commercial plates according to Decision 2020/1729/EU [26].

Two sensititre susceptibility panels were used to test isolates in order to phenotypically categorize presumed ESBL, AmpC, and carbapenemase producers (Thermo Fisher Scientific, Waltham, MA, USA). Ten classes (Ampicillin, Azithromycin, Cefotaxime, Chloramphenicol, Ciprofloxacin, Col-istin, Gentamicin, Meropenem, Nalidixic acid, Sulphametoxasole, Ceftazidime, Tetracyline, Tigecycline, Trimethoprim) of antimicrobial drugs were represented in the first panel, EUVSEC1. EUVSEC2, the second panel, included ten antimicrobial substances: Ertapenem, Cefepime, Cefotaxime, Cefotaxime with Clavulanic acid, Cefoxitin, Imipenem, Meropenem, Ceftazidime, Ceftazidime with Clavulanic acid, and Temocillin. A 50 μL inoculum from Mueller–Hinton Broth of 1 × 10^5^ cfu/mL was inoculated in 96 wells of commercial microtiter panels EUVSEC1 and EUVSEC2. The panels were incubated at 35–37 °C for 16–18 h. The findings were observed following incubation of plates at 35–37 °C for 16–18 h. Epidemiological cutoff values (ECOFFs) provided by the European Committee for Antimicrobial Susceptibility Testing (EUCAST) were used to interpret MIC results in order to define resistance to the tested antibiotic. The synergy, which is necessary for the phenotypic classification of ESBL and/or AmpC synthesis, was tested using clavulanic acid.

### 2.6. Analytical Standards

The analytical standards were supplied from Sigma-Aldrich (St. Louis, MO, USA) (amoxicillin (99.6%), ampicillin (99.8%), procaine benzylpenicillin (99.3%), cloxacillin (98.7%), oxacillin (98.4%), lincomycin (100.3%), tylosin (87.9%), trimethoprim (99.5%), tetracycline (96.8%), cefapirin (98.5%)) and Dr Ehrenstorfer GmbH (Augsburg, Germany) (ceftiofur (98.01%), cefalexin (96.6%), oxytetracycline (96.5%), enrofloxacin (99.74%), ciprofloxacin (98.0%), sulfadimidine (99.6%), sulfamethoxazole (99.7%), sulfadiazine (99.8%), sulfachlorpyridazine (99.1%) and sulfadimethoxine (99.7%)).

### 2.7. Isotopically Labelled Internal Standards

Flunixin-d3 (100.0%) and penicillin G-d7 *N*-ethyl-piperidinium salt (98.1%) were obtained from Sigma-Aldrich (St. Louis, MO, USA).

### 2.8. Preparation of Stock, Intermediate and Working Standard Solutions

Methanol was used to make stock solutions for individual and internal standards that range in concentration from 0.5 mg/mL to 1.0 mg/mL. A mixture of 10 μg/mL of working solutions from standards and internal standards was made in methanol. Blank bovine feces and blank milk samples were used to create matrix-matched calibration curves. The blank feces and milk samples were homogenized for 5 min on a rotary shaker before fortification with the antimicrobial standards.

### 2.9. Chemicals and Reagents

LC-MS/MS grade water, acetonitrile, methanol, HPLC grade ammonium acetate, Na_2_EDTA (p.a.) and disodium hydrogen phosphate dihydrate (p.a.) were obtained from Carlo Erba Reagent S.A.S (Val de Reuil, France). The purchase of formic acid (LC-MS grade), citric acid (99.0%) and trichloroacetic acid (99.5%) was made at Merck (Darmstadt, Germany). Oasis HLB (hydrophilic-lipophilic balanced) 6 cc vac cartridges with 500 mg sorbent per cartridge were procured by Waters (Milford, MA, USA).

In the preparation of McIlvaine-Na_2_EDTA buffer, 115.65 mL 0.2 M phosphate buffer and 184.65 mL 0.1M citric acid were used to dissolve 11.406 g Na2EDTA. To adjust the pH to 4.0, 0.1 M citric acid or 0.2 M phosphate buffer were added. The phosphate solution (0.2 M) was prepared by dissolving 31.2 g Na_2_HPO_4_·2H_2_O in 1 L of water. Citric acid (0.1 M) solution was prepared by dissolving 19.2 g of citric acid in 1000 mL of water.

### 2.10. Sample Preparation

The used method was a modification of previous work by Patyra et al. (2020) [27] and Pokrant et al. (2020) [28], but we validated it according to 2002/657/EC [29]. The feces samples were homogenized and 2.0 g accurately weighed and placed in 50 mL plastic tubes. The mixture was shaken on a vortex machine for 1 min and kept for 3 h for equilibration in a dark place. For extraction of the antimicrobial residues, 15 mL of Na_2_EDTA- McIlvaine buffer and 5 mL acetonitrile were added to the samples, which were then shaken for 45 min on a horizontal shaker and after that, were centrifuged at 4000 rpm for 20 min at room temperature. The supernatant was taken, and the extraction procedure was repeated once more. The two supernatants were combined, followed by solid phase extraction with Oasis HLB cartridges (500 mg/6 mL). Before loading and running the supernatant through the Oasis HLB cartridges, the cartridges were preconditioned with 5 mL of methanol and 5 mL of water. After running the supernatant, the cartridges were rinsed with 5 mL of water and dried 20 min using a manifold pump. 5 mL methanol was used to extract the residues from the columns and the eluate was evaporated in a 35 °C water bath with a gentle flow of nitrogen. Before LC-MS/MS analysis, the residues from antibiotics in the samples were dissolved in 1 mL of the mobile phase and filtered through a 0.45 µm syringe filter.

A 5 mL aliquot of milk was placed in a 50 mL plastic centrifuge tube to prepare the milk samples. 20% aqueous trichloroacetic acid was added at a volume of 2 mL. For five minutes, the samples were shaken. 20 mL of McIlvaine buffer were added after shaking. The samples were centrifuged at +4 °C for 20 min at 4000 rpm after being vortexed for 1 min. The SPE cartridge was immediately loaded with the supernatant. Earlier, the cartridge was activated using 3 methanol and 2 mL of water. Following sample loading, the cartridge was cleaned with 4 mL of water and fully vacuum dried for 20 min. Three milliliters of methanol were used to elute antibiotic residues. The samples were dried by being evaporated to dryness in a nitrogen stream at 35 °C. After being reconstituted in 250 μL of mobile phase (98% mobile phase A and 2% mobile phase B), the dry residues were filtered through a 0.22 μm microfilter. The final extract was injected into the LC-MS/MS apparatus.

### 2.11. LC-MS/MS Analysis

A LC-MS/MS instrument purchased from Waters (Milford, MA, USA) was used to determine the antimicrobial residues in milk and feces. The LC-MS/MS instrument consisted of an autosampler (thermostatted), column manager, vacuum degasser, binary pump and triple quadrupole detector. A Kinetex C18 column (50 mm × 2.1 mm, 2.6 μm) obtained from Phenomenex (Torrance, CA, USA) was used for the chromatographic separation of antibiotic residues. The LC-MS/MS system was managed by the MassLynx software (version 4.1, Waters), which was also utilized to process the analytical results.

The LC-MS/MS conditions were as previously reported [30]. Briefly, 5 μL of injection volume was used, with a flow rate of 0.2 mL/min, at a column temperature of 40 °C. To separate the antimicrobial residues, the following gradient elution protocol was used: 0–1 min 95%–80% A; 1–4 min 80%–60% A; 4–8 min 60%–0% A; 8–10 min 0% A; 10–10.3 min 0–95% A and 10.3–12 min 95% A. From the six mobile phases that were tested and reported by Hajrulai-Musliu et al. [30], mobile phase A (aqueous 5 mM ammonium acetate, 0.01% formic acid and 0.01% trichloroacetic acid) and mobile phase B (methanol with 0.1% formic acid) were chosen as the optimal mobile phases. Capillary voltage of 3.0 kV, source temperature of 150 °C, desolvation temperature of 400 °C, cone gas of 100 L/h, and desolvation gas of 300 L/h were the ideal MS/MS parameters. The electrospray positive mode (ESI+) was used along with multiple reaction monitoring (MRM) of two transition ions which along with the retention times facilitated identification of the antimicrobial residues.

### 2.12. Method Validation

The method was validated according to the Commission Decision 2002/657/EC [29] and the International Council for Harmonization of Technical Requirements for Pharmaceuticals for Human Use Q2 (R1) guidelines [31]. The parameters validated were linearity, specificity/selectivity, limit of detection (LOD), limit of quantification (LOQ), decision limit (CCα), detection capability (CCβ), recovery and precision. For linearity matrix-matched calibration, curves in the range from LOQ to 1000 µg/kg were prepared by spiking blank feces and milk samples with the standard solutions. Specificity/selectivity of the method was assessed through the analysis of 20 blank feces samples. The LOD was obtained as the mean value of the lowest concentration of the standard curve (*n* = 6) plus 3.3 times the calculated standard deviation (SD), while the LOQ was the lowest concentration plus 10 times the calculated SD. The CCα and CCβ were calculated using the criteria prescribed in European Commission Decision 2002/657/EC [29]. Instead of trueness, the recovery was conducted, because certified reference material was not available. For evaluation of the recovery, intra-day and inter-day precision, feces samples were spiked at three concentration levels in six replicates each. For the inter-day precision, the study was repeated on three consecutive days. The calculated concentrations were compared with fortified concentration and recoveries calculated as percentage values. The standards deviations (SD) and coefficient of variation (CV) were used to evaluate intra-day and inter-day precisions.

## 3. Results

### 3.1. Detection and Quantification of Antimicrobial Residues in Milk and Feces Samples

#### 3.1.1. LC–MS/MS Optimization

The LC–MS/MS method was used for the simultaneous determination of 20 antibiotic residues in feces and milk samples.

The MS conditions used to monitor each transition are summarized in Table 1. Stock solutions of individual drugs at 1 µg/mL were analyzed to verify MRM transitions and the retention time (*t*_R_) selected.

#### 3.1.2. Linearity of the Method

The linearity of the calibration curve of the antibiotic standards used in the study was evaluated by calculating the coefficient of correlation (R^2^). The results obtained (Table 2) about the R^2^ were in the range from 0.9929 to 0.9999 for feces and from 0.9951 to 0.9999 for milk. R^2^ was satisfactory for all the antimicrobial standards used in the study.

#### 3.1.3. Selectivity

The method demonstrated good selectivity as no interferences were observed at the retention times of the target analytes in the 10 blank samples.

#### 3.1.4. Limit of Detection (LOD), Limit of Quantification (LOQ), Decion Limit (CCα) and Detection Capability (CCβ)

For feces samples, the LOD and LOQ ranged from 8.26 µg/kg to 27.60 µg/kg and 21.17 µg/kg to 59.60 µg/kg, respectively, while the CCα and CCβ were within the range of 15.35–51.48 µg/kg and 25.10–66.41 µg/kg, respectively.

For milk samples, the LOD and LOQ ranged from 1.28 µg/L to 47.94 µg/L and 1.76 µg/L to 55.78 µg/L, respectively, while the CCα and CCβ were within the range of 4.66–179.19 µg/L and 5.23–199.07 µg/L, respectively (Table 3).

#### 3.1.5. Accuracy and Precision of the Method

The recovery, repeatability (intra-day precision) and reproducibility (inter-day precision) values are shown in Table 4.

For the feces samples, the recovery ranged from 80.07% to 105.92%; the coefficient of variation (CV) for repeatability ranged from 0.80% to 11.06%, while the CV for reproducibility ranged from 2.58% to 14.99%. The chromatograms from spiked feces samples at concentration level 100 μg/kg are given as Supplementary Materials (Figure S1).

For the milk samples, the recovery ranged from 70.83% to 109.00%; the coefficient of variation (CV) for repeatability ranged from 2.41% to 21.12%, while the CV for reproducibility ranged from 3.48% to 23.44%. The chromatograms from spiked milk samples are given as Supplementary Materials (Figure S2).

The concentrations of antimicrobials oxytetracycline, enrofloxacin, amoxicillin, trimethoprim and procaine-benzylpenicillin were quantified in both milk and feces at each of the sampling days (Table 5). All administered antimicrobials showed the highest concentrations, above respective MRLs (100 µg/kg for enrofloxacin and oxytetracycline; 50 µg/kg for trimethoprim, and 4 µg/kg for amoxicillin and procaine benzylpenicillin), on the first day after drug administration and then they gradually decreased until were eliminated after 7th day of the treatment.

On the third day during the withdrawal period, slightly higher values than their MRLs were noted for procaine benzylpenicillin (4.4 µg/L in milk and 24.88 µg/kg in feces samples) and for amoxicillin residues (27.11 µg/L in milk samples). Concentrations of other residues in analyzed milk and feces samples were below their MRL values (Table 5).

In this study, concentrations of antimicrobial residues have shown a sharp decrease. The concentration of residues was higher in milk samples compared with feces. The exception was amoxicillin residues.

**Figure 1:**
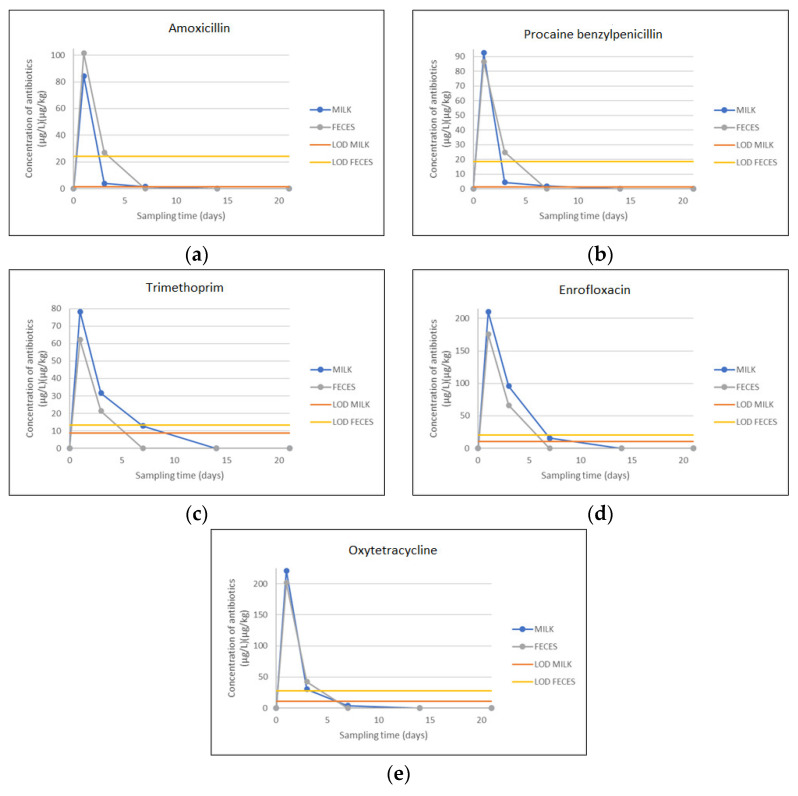
The residue depletion curves for both groups of samples (milk and feces). (**a**) Amoxicillin residues in milk and feces samples obtained from cattle after single-dose administration with Amoxicillin 150 mg/mL; (**b**) Procaine benzylpenicillin residues in milk and feces samples obtained from cattle after single-dose administration with procaine-benzyl penicillin G 200,000 IU/mL; (**c**) Trimethoprim residues in milk and feces samples obtained from cattle after single-dose administration with Trimethoprim 80 mg/mL; (**d**) Enrofloxacin residues in milk and feces samples obtained from cattle after single-dose administration with Enrofloxacin 100 mg/mL; (**e**) Oxytetracycline residues in milk and feces samples obtained from cattle after single-dose administration with Oxytetracycline 200 mg/mL.

The residue depletion curves for both groups of samples (milk and feces) are shown in Figure 1.

### 3.2. Isolation of Presumptive ESBL/AmpC- and Carbapenemase-Producing E. coli from Feces Samples

Isolation of ESBL-, AmpC- and carbapenemase-producing *E. coli* from feces samples was performed using an established protocol (Commission Implementing Decision (EU) 2020/1729). This protocol is able to detect resistant isolates through pre-enrichment phase and inoculation on different selective and chromogenic agar plates.

The phenotypic characterization of commensal *E. coli* in all of the analyzed feces samples was determined thought VITEK 2 Compact System.

When selective MacConkey agar was inoculated with Cefotaxime, five (25%) presumptive ESBL/AmpC-generating strains of *E. coli* were found; however, organisms that produced carbapenemases were not identified when tested on a bi-plate selective chromogenic medium.

### 3.3. Antimicrobial Susceptibility Test (AST) Determination by Broth Microdilution

The broth microdilution method was used for antimicrobial susceptibility testing, using two commercial antibiotic panels, EUVSEC1 and EUVSEC2.

**Figure 2:**
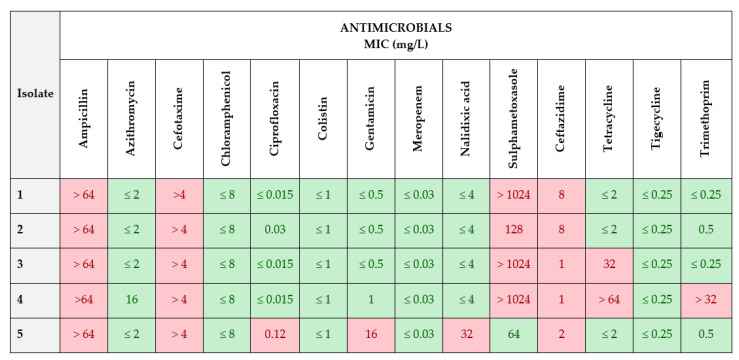
MIC values (mg/L) on EUVSEC1 commercial panel. Pink squares—resistant isolate (R); Green squares—susceptible isolates (S).

The results of MIC values of the first commercial panel EUVSEC1 are shown in Figure 2. The protocol allows growth of only *E. coli* resistant to cephalosporin. The results of inoculation of the samples on chromogenic, selective MacConkey agar with Cefotaxime showed that all isolates are resistant to cefotaxime, ampicillin and ceftazidime. The MIC value for ampicillin in all five isolates was above 64 mg/L and for cefotaxime was above 4 mg/L. MIC value for ceftazidime in isolates 1 and 2 was 8 mg/L, in isolates 3 and 4 was 1 mg/L, and in isolate 5 was 2 mg/L (Figure 2).

**Figure 3:**
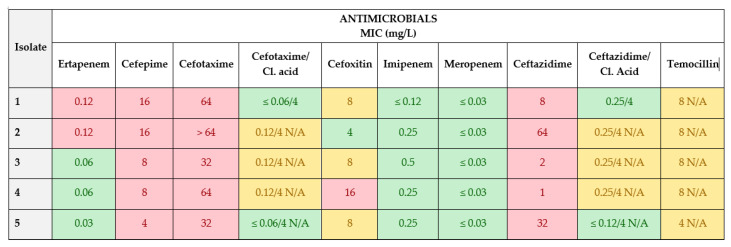
MIC values (mg/L) on EUVSEC2 commercial panel. Pink squares—resistant isolate (R); Green squares—susceptible isolates (S); Yellow squares—N/A (nonapplicable).

Isolates in which MIC values for cefotaxime and ceftazidime antimicrobials were >1 mg/L were subjected to further phenotypic characterization with a second plate EUVSEC2 (Figure 3).

The MIC values of EUVSEC2 antibiotic panel have shown resistance of isolates to antimicrobials: ertapenem, cefepime, cefotaxime, cefixime and ceftazidime. Isolates 1 and 2 showed resistance to ertapenem with MIC of 0.12 mg/L. All five isolates were resistant to cefepime. Their MIC values were 16 mg/L (1 and 2 isolate), 8 mg/L (3 and 4 isolate) and 4 mg/L (isolate 5). For cefotaxime, antimicrobials recorded high MIC values of 64 mg/L (1, 2 and 4 isolate) and 32 mg/L (3 and 5 isolate). Only one isolate (4) was resistant to cefoxitin with the MIC value of 16 mg/L. The MIC values for ceftazidime were 8 mg/L, 64 mg/L, 2 mg/L, 1 mg/L and 32 mg/L for isolates number 1, 2, 3, 4 and 5, respectively.

Interpretation of phenotypic profiles for determination of resistant ESBL and AmpC *E. coli* was based on EUCAST guidelines for the detection of resistance mechanisms and specific resistances of clinical and/or epidemiological importance [32]. Briefly, an ESBL phenotype is determined if isolates are resistant to cefotaxime (>1 mg/L) or ceftazidime (>1 mg/L) but susceptible to cefoxitin (≤8 mg/L) and they show clavulanic acid synergy with cefotaxime and/or ceftazidime (≥8-fold reduction of MIC of the cephalosporin combined with 4 mg/L clavulanic acid compared with the MIC of the cephalosporin alone). Isolates are considered to have the AmpC phenotype if they do not show clavulanic acid synergy and are resistant to cefotaxime (>1 mg/L) or ceftazidime (>1 mg/L) and cefoxitin (>8 mg/L). An ESBL+AmpC phenotype is determined if isolates are resistant to cefotaxime (>1 mg/L) or ceftazidime (>1 mg/L), resistant to cefoxitin (>8 mg/L), and they show clavulanic acid synergy with cefotaxime and/or ceftazidime.

In this study, according EUCAST guidelines, four isolates of commensal *E coli* (1, 2, 3 and 5) were determined as ESBL phenotype and only one (isolate number 4) was determined as ESBL+ AmpC phenotype.

## 4. Discussion

Livestock and their surroundings appear as significant reservoirs of resistant bacteria, which is a result of the extensive and frequent administration of antimicrobials [33].

There are numerous screening and confirmatory methods for determination of antibiotic residues in milk and tissues. Liquid chromatography combined with tandem mass spectrometry (LC–MS/MS method) is the most frequently applied confirmatory method. This technique enables the identification and determination of multiple classes of antibiotics in milk and tissues [34].

In this study, the LC–MS/MS method was used for simultaneous determination of antimicrobial residues of oxytetracycline, enrofloxacin, amoxicillin, trimethoprim and procaine benzylpenicillin. A closely related method previously developed was used for the detection of 23 veterinary drugs from six different groups in bovine milk [35]. Accuracy and precision of the method comply with the criteria prescribed in Commission Decision 2002/657/EC [29]. According to the validation results, it can be concluded that the applied method is suitable for simultaneous analysis of multi-class and multi-residue antimicrobials in milk and feces samples.

Obtained results from the detection and quantification of antimicrobial residues in milk and feces samples show a sharp decrease in concentrations of antimicrobial residues. Their concentrations started to decrease after the first day of drug administration, and they were eliminated after the 7th day of the treatment. Previous studies have shown similar patterns in residue depletion [36,37]. The minimum withdrawal period for oxytetracycline is 3 days [38], for amoxicillin is 3 days [39] for procaine benzylpenicillin is 5 days [40], for enrofloxacin is 3 days and for trimethoprim is 3.5 day [41].

According to results obtained for MIC values and phenotypic determination of commensal *E. coli* isolates based on commercial antimicrobial panels, it is important to underline the fact that ESBL-producing *E. coli* isolates in this study have shown high resistance toward cephalosporins, to the third and fourth generations. Also, high resistance was determined for sulfamethoxazole (SULM) as part of the combined drug therapy of the sulfamethoxazole/trimethoprim that was used during this experiment. No high MIC values were registered toward antimicrobials used in this experiment or to other antimicrobials which were part of commercial plates. Although ertapenem resistance was detected, according to the phenotypic categorization of the isolates, carbapenem phenotype was not determined.

Obtained results in this study are in line with other reported findings from similar studies worldwide. Very similar rates of antimicrobial resistance patterns of *Escherichia coli* isolates were determined in dairy cattle from small-scale farming systems in Tanzania where the resistant rate to ampicillin and cefotaxime was 96.7%, and 95%, respectively [42], and by Kijima-Tanaka et al. in Japan [43] and Bywater [44] in the European Union determined the rate was about 30%. Meanwhile, compared to other findings in this study, different results were obtained regarding the level of resistance. A study from Korea determined the resistance level was in a range between 0.6–11.7% for cefoxitin and penicillin [45]. In a study from the United Kingdom, the prevalence of resistant *E. coli* isolates in different cattle groups was as follows: heifers 2.6%, dry cows 3.4% and low-yield milking cows 31.2% [46].

Numerous studies have reported a high prevalence of ESBL-producing *E. coli* in environmental samples, especially from farm environments. The findings and supporting data imply that dairy cattle farms are a substantial source of ESBL-producing *E. coli* that play an important role in resistance dissemination across the ‘One Health’ concept [47,48,49].

This type of experiment, which includes a single treatment of animals with different antimicrobials, may have a direct influence or can be related to the emergence or dissemination of antimicrobial resistance between farm animals or into the environment around farms.

Promotion of proper use of antimicrobials in livestock and frequent monitoring play an important role in determination of antimicrobial resistance. The applied LC–MS/MS method together with performed analysis of residue depletion curves in analyzed samples can contribute to the understanding of antimicrobial resistance in food-producing animals and the environment.

## 5. Conclusions

This study aimed to determine the antimicrobial residue levels in milk and feces samples during the withdrawal period in dairy cattle administrated with a single dose of the drug, as well as to characterize the antimicrobial resistance patterns of commensal isolates of *Escherichia coli* cultured from cattle feces samples.

The concentrations of antimicrobials oxytetracycline, enrofloxacin, amoxicillin, trimethoprim and procaine benzylpenicillin were quantified in both milk and feces at each of the sampling days. All administered antimicrobials showed the highest concentrations, above their MRLs values, on the first day after drug administration and then they gradually decreased until their elimination (after the 7th day of the treatment).

Antimicrobial pressure on the microbiome in animals or the environment is the result of pressure during a long period of time, through which bacteria ensure their survival, but, obviously, misuse and overuse of semi-synthetic and synthetic antimicrobials play the leading and key role of emerging and dissemination of antimicrobial resistance through bacterial mechanisms.

Regular monitoring would enable timely identification of both emerging and existing forms of antimicrobial resistance in food-producing animals.

## Figures and Tables

**Table 1:** MRM (Multiple Reaction Monitoring) parameters for each of 20 antimicrobials and two internal standards.

Standard	Ionisation Mode (ESI)	Precursor Ion (m/z)	Product Ion (m/z)	Collision Energy	Cone Voltage	Retention Time
Amoxicillin	+	367.07	159.96	16	28	5.55
			90.89	40		
Ampicillin	+	349.97	159.94	14	34	3.93
			105.95	20		
Procaine benzylpenicillin	+	334.99	90.96	42	44	5.52
			80.94	52		
Lincomycin	+	407.06	126.02	34	22	2.80
			41.75	72		
Tylosin	+	916.30	100.88	52	74	6.31
			173.99	46		
Trimethoprim	+	290.97	229.94	24	26	2.90
			122.94	28		
Cephapirin	+	423.93	291.93	14	42	2.04
			151.89	28		
Tetracycline	+	445.03	410.01	20	40	5.33
			153.90	34		
Cloxacillin	+	435.94	159.97	18	26	6.15
			276.96	14		
Oxacillin	+	402.05	159.96	10	24	5.95
			243.03	12		
Cefalexin	+	347.97	173.93	14	30	2.75
			157.86	8		
Ceftiofur	+	523.96	125.17	58	34	4.90
			241.00	16		
Enrofloxacin	+	360.05	72.02	36	36	3.68
			245.09	30		
Ciprofloxacin	+	332.01	230.94	28	38	3.56
			245.05	40		
Oxytetracycline	+	462.01	426.02	38	36	3.17
			200.93	30		
Sulfachlorpyridazin	+	284.9	155.93	16	28	2.93
			91.93	34		
Sulfadiazine	+	250.97	155.93	14	28	1.92
			91.93	30		
Sulfadimethoxine	+	310.97	91.93	32	36	4.36
			155.93	20		
Sulfadimidine	+	278.95	91.93	36	34	2.71
			185.93	18		
Sulfamethoxazole	+	253.91	155.94	16	28	3.01
			92.00	30		
Flunixin–D3	+	300.03	263.98	36	25	6.80
Penicillin G–d7	+	374.03	159.94	16	32	5.51

**Table 2:** Linearity of the method.

Standard	Calibration Range		R^2^	
	Matrix		Matrix	
	Feces	Milk	Feces	Milk
	(μg/kg)	(μg/L)		
Amoxicillin	20.0–1000	1.0–50	0.9998	0.9993
Ampicillin	20.0–1000	1.0–50	0.9989	0.9963
Procaine benzylpenicillin	20.0–1000	1.0–50	0.9997	0.9999
Lincomycin	20.0–1000	50.0–300	0.9984	0.9978
Tylosin	20.0–1000	10.0–100	0.9978	0.9991
Trimethoprim	20.0–1000	10.0–100	0.9997	0.9994
Cephapirin	20.0–1000	10.0–100	0.9999	0.9994
Tetracycline	20.0–1000	10.0–200	0.9944	0.9974
Cloxacillin	20.0–1000	10.0–100	0.9961	0.9954
Oxacillin	20.0–1000	10.0–100	0.9984	0.9951
Cefalexin	20.0–1000	10.0–200	0.9929	0.9976
Ceftiofur	20.0–1000	10.0–200	0.9937	0.9979
Enrofloxacin	20.0–1000	10.0–200	0.9999	0.9981
Ciprofloxacin	20.0–1000	10.0–200	0.9949	0.9997
Oxytetracycline	20.0–1000	10.0–200	0.9994	0.9963
Sulfachlorpyridazin	20.0–1000	10.0–200	0.9974	0.9967
Sulfadiazine	20.0–1000	10.0–200	0.9979	0.9958
Sulfadimethoxine	20.0–1000	10.0–200	0.9979	0.9970
Sulfadimidine	20.0–1000	10.0–200	0.9981	0.9988
Sulfamethoxazole	20.0–1000	10.0–200	0.9978	0.9994

**Table 3:** Limit of detection (LOD), Limit of quantification (LOQ), Decision limit (CCα) and Detection capability (CCβ).

Standard	LOD		LOQ		CCα		CCβ	
	Matrix		Matrix		Matrix		Matrix	
	Feces	Milk	Feces	Milk	Feces	Milk	Feces	Milk
	(μg/kg)	(μg/L)	(μg/kg)	(μg/L)	(μg/kg)	(μg/L)	(μg/kg)	(μg/L)
Amoxicillin	24.10	1.59	32.11	2.59	42.36	5.34	52.11	6.43
Ampicillin	20.18	1.28	57.17	1.76	35.14	4.66	49.78	5.23
Procaine benzylpenicillin	18.33	1.36	28.65	2.16	43.18	4.80	56.71	5.66
Lincomycin	14.10	47.94	35.17	55.78	27.45	179.19	37.52	199.07
Tylosin	12.80	10.71	34.12	13.62	20.12	55.31	31.40	66.09
Trimethoprim	13.36	8.68	21.17	11.01	38.29	54.74	49.40	65.31
Cephapirin	8.26	12.33	23.01	15.72	21.35	71.91	32.14	82.65
Tetracycline	21.35	8.65	55.60	11.05	41.78	105.91	52.40	116.86
Cloxacillin	22.18	10.24	59.60	12.97	37.54	31.74	54.25	35.93
Oxacillin	17.15	9.26	41.10	14.01	30.12	35.12	41.35	41.32
Cefalexin	16.35	12.38	35.50	15.02	21.35	105.14	34.17	118.36
Ceftiofur	9.12	11.22	26.40	14.67	15.35	101.66	27.14	108.76
Enrofloxacin	20.40	10.53	35.42	14.12	47.80	112.33	59.71	132.14
Ciprofloxacin	23.14	12.80	55.35	16.60	39.14	102.12	57.80	121.14
Oxytetracycline	27.60	10.76	41.60	12.12	51.48	113.65	66.41	131.29
Sulfachlorpyridazin	14.35	10.47	32.18	13.88	22.15	116.82	40.78	142.59
Sulfadiazine	21.30	10.89	55.60	13.39	27.15	121.95	58.35	152.05
Sulfadimethoxine	12.25	10.35	31.48	12.69	17.14	104.70	25.10	122.17
Sulfadimidine	12.70	9.06	34.15	11.42	19.48	121.30	31.40	146.93
Sulfamethoxazole	15.30	10.13	48.12	13.03	23.54	103.81	37.80	119.54

**Table 4:** Accuracy and precision of the method.

Standards	Added		Average Concentration in the Samples (*n* = 6)		Standard Deviation		Recovery (%)		Repeatability		Reproducibility	
	Concentration				(μg/kg)				(CVr, %)		(CV_R_, %)	
	Matrix		Matrix		Matrix		Matrix		Matrix		Matrix	
	Feces	Milk	Feces	Milk (μg/L)	Feces	Milk (μg/L)	Feces	Milk	Feces	Milk	Feces	Milk
	(μg/kg)	(μg/L)	(μg/kg)		(μg/kg)							
Amoxicillin	100	2	89.00	2.18	3.81	0.24	89.00	109.00	4.28	10.99	10.69	19.37
	250	4	235.71	4.26	10.38	0.66	94.28	106.38	4.40	15.59	9.02	18.90
	500	6	464.26	5.22	17.66	0.61	92.85	86.97	3.80	11.61	14.37	14.57
Ampicillin	100	2	80.07	1.74	3.13	0.21	80.07	87.51	3.90	12.07	11.35	16.64
	250	4	238.59	3.56	11.08	0.65	95.44	89.36	4.64	18.26	14.88	20.18
	500	6	412.29	5.71	12.18	1.03	82.46	95.17	2.96	18.04	10.43	21.35
Procaine benzylpenicillin	100	2	83.48	1.42	6.37	0.14	83.48	70.83	7.62	9.53	10.48	15.99
	250	4	243.11	3.92	13.61	0.53	97.24	97.92	5.60	13.59	6.17	18.18
	500	6	473.10	4.93	17.87	0.35	94.62	82.08	3.78	7.10	6.60	14.23
Lincomycin	100	75	85.56	61.25	6.00	3.14	85.56	81.67	7.01	5.13	14.22	6.82
	250	150	230.61	159.31	12.28	12.12	92.24	106.20	5.32	7.61	12.76	8.87
	500	225	428.11	218.62	10.78	27.88	85.62	97.16	2.52	12.76	6.16	15.78
Tylosin	100	25	86.39	22.25	4.63	2.30	86.69	89.01	5.36	10.32	10.94	14.72
	250	50	204.21	44.53	16.96	6.57	81.68	89.07	8.30	14.76	14.99	17.14
	500	75	488.40	74.53	8.28	4.82	97.68	99.38	1.70	6.47	5.68	10.84
Tylosin	100	25	97.11	21.58	5.64	2.79	97.11	86.31	5.80	12.94	13.03	15.85
	250	50	263.79	44.17	12.02	6.44	105.92	88.34	4.56	14.59	8.03	17.37
	500	75	524.30	73.07	19.47	6.95	104.86	97.43	3.71	9.51	11.10	11.73
Cephapirin	100	30	97.74	26.26	4.99	1.23	97.74	87.52	5.11	4.68	10.36	7.31
	250	60	259.89	61.18	12.12	6.54	103.96	101.96	4.66	10.7	14.85	13.06
	500	90	458.11	82.28	25.58	1.99	91.62	91.42	5.58	2.41	12.10	3.48
Tetracycline	100	25	102.32	44.78	7.20	3.82	102.32	89.57	7.04	8.53	12.55	12.52
	250	50	230.34	94.95	12.22	6.68	92.14	94.95	5.30	7.04	11.39	9.80
	500	75	491.56	126.81	11.55	8.30	98.31	84.54	2.35	6.55	8.18	8.41
Cloxacillin	100	15	105.4	14.83	3.77	1.21	105.4	98.88	3.58	8.19	6.77	10.74
	250	30	255.39	27.54	4.95	2.56	102.16	91.81	1.94	9.28	3.98	12.12
	500	45	488.92	39.51	11.80	4.48	97.78	87.80	2.41	11.33	5.89	17.86
Oxacillin	100	15	89.29	14.22	4.92	2.44	89.29	94.84	5.51	17.16	9.66	22.04
	250	30	221.37	27.36	7.98	3.08	88.55	91.22	3.61	11.26	6.01	14.33
	500	45	450.84	40.17	6.83	5.44	90.17	89.27	1.23	13.54	4.44	16.28
Cefalexin	100	50	85.73	41.48	5.14	5.26	85.73	82.96	6.00	12.68	7.61	15.87
	250	100	244.44	88.48	7.19	7.11	97.77	88.48	2.94	8.04	8.21	10.97
	500	150	484.13	123.17	5.93	9.22	96.83	82.11	1.93	7.49	7.33	10.02
Ceftiofur	100	50	93.60	50.49	2.13	3.67	93.6	100.97	2.28	7.27	5.73	14.39
	250	100	246.23	94.57	9.15	4.33	98.49	94.57	3.72	4.57	5.12	7.82
	500	150	480.64	132.32	8.85	9.27	96.13	88.21	1.84	7.00	4.30	9.89
Enrofloxacin	100	50	82.64	54.10	8.60	7.47	82.64	108.2	10.41	13.8	13.31	15.92
	250	100	212.54	92.52	12.42	12.08	85.01	92.52	5.84	13.06	14.81	14.25
	500	150	432.07	141.36	27.74	17.48	86.41	98.22	6.42	11.86	14.74	14.96
Ciprofloxacin	100	50	89.56	45.49	3.24	3.14	89.56	90.97	3.62	6.91	8.76	9.73
	250	100	247.88	83.10	4.79	11.6	99.15	83.10	1.93	13.96	5.61	15.46
	500	150	495.32	131.55	3.98	16.46	99.06	87.70	0.80	12.51	2.11	15.98
Oxytetracycline	100	50	89.82	40.68	9.93	3.28	89.82	88.04	11.06	7.45	14.99	9.39
	250	100	209.99	96.00	14.24	10.76	83.99	96.00	6.78	11.21	14.76	13.22
	500	150	421.17	159.94	21.69	13.38	84.23	106.63	5.15	8.36	12.98	10.91
Sulfachlorpyridazin	100	50	82.45	48.06	2.52	7.51	82.45	96.11	3.06	15.63	5.21	18.96
	250	100	219.02	91.05	4.01	15.71	87.61	91.05	1.83	17.26	4.68	19.98
	500	150	470.61	151.33	11.69	18.25	94.12	100.89	2.48	12.06	5.02	13.33
Sulfadiazine	100	50	86.08	46.89	4.51	8.74	86.08	93.79	5.25	18.63	6.90	21.68
	250	100	246.59	91.85	6.09	18.35	98.63	91.85	2.47	19.98	3.77	20.84
	500	150	488.60	141.33	8.44	7.33	97.72	94.21	1.73	5.18	2.58	8.18
Sulfadimethoxine	100	50	83.62	42.42	1.67	4.86	83.62	84.84	2.00	11.46	3.09	14.55
	250	100	226.68	87.23	7.03	10.65	90.67	87.23	3.10	12.21	6.20	13.34
	500	150	467.95	123.81	13.21	12.21	93.59	82.54	2.82	11.00	6.62	13.30
Sulfadimidine	100	50	97.40	42.50	2.75	8.98	97.40	84.99	2.83	21.12	6.29	23.44
	250	100	243.62	95.66	5.60	15.63	97.45	95.66	2.30	16.33	4.03	17.22
	500	150	511.71	132.17	5.54	20.01	102.34	88.11	1.08	15.14	2.61	16.77
Sulfamethoxazole	100	50	85.05	46.38	2.93	5.80	85.05	92.75	3.45	12.51	8.23	16.53
	250	100	236.86	88.06	11.12	9.60	94.74	88.06	4.69	10.90	6.14	13.27
	500	150	480.43	120.05	5.77	11.45	96.09	80.03	1.08	9.54	2.61	13.70

**Table 5:** Concentration of antimicrobials (µg/L or µg/kg) in milk and feces analyzed by LC-MS/MS.

Antibiotic	Matrix	Sampling Days								
		0	1	3	7	14	21	LOD	CCα	MRL
Amoxicillin	Milk	ND	84.35	3.80	1.35	<LOD	<LOD	1.28	5.34	4.0
	µg/L									
	Feces	ND	101.38	27.11	<LOD	<LOD	<LOD	24.10	42.36	/
	µg/kg									
Procaine benzylpenicillin	Milk	ND	92.50	4.40	1.72	<LOD	<LOD	1.36	4.80	4.0
	µg/L									
	Feces	ND	86.30	24.88	<LOD	<LOD	<LOD	18.33	43.18	/
	µg/kg									
Trimethoprim	Milk	ND	78.30	31.60	12.80	<LOD	<LOD	8.68	54.74	50.0
	µg/L									
	Feces	ND	62.18	21.30	<LOD	<LOD	<LOD	13.36	38.29	/
	µg/kg									
Enrofloxacin	Milk	ND	210.10	95.60	15.40	<LOD	<LOD	10.53	112.33	100.0
	µg/L									
	Feces	ND	175.48	66.20	<LOD	<LOD	<LOD	20.40	47.80	/
	µg/kg									
Oxytetracycline	Milk	ND	221.10	30.20	3.90	<LOD	<LOD	10.76	113.65	100.0
	µg/L									
	Feces	ND	201.30	42.45	<LOD	<LOD	<LOD	27.60	51.48	/
	µg/kg									

## Data Availability

The data presented in this study are available on request from the corresponding author.

## Supplementary Materials

The following supporting information can be downloaded at: https://www.mdpi.com/article/10.3390/ani13233603/s1, Figure S1: Chromatograms of spiked feces samples and Figure S2: The chromatograms of spiked milk samples.
