# Supplementary material for: Determination of Multi-Class Antimicrobial Residues and Antimicrobial Resistance in Cow Milk and Feces Samples during Withdrawal Period
Source: Animals (Basel). 2023 Nov 22;13(23):3603. doi: 10.3390/ani13233603 (PMC10705613; doi:10.3390/ani13233603)
Supplement: Supplementary file 1 [file animals-13-03603-s001.zip › animals-2637269-supplementary.pdf]

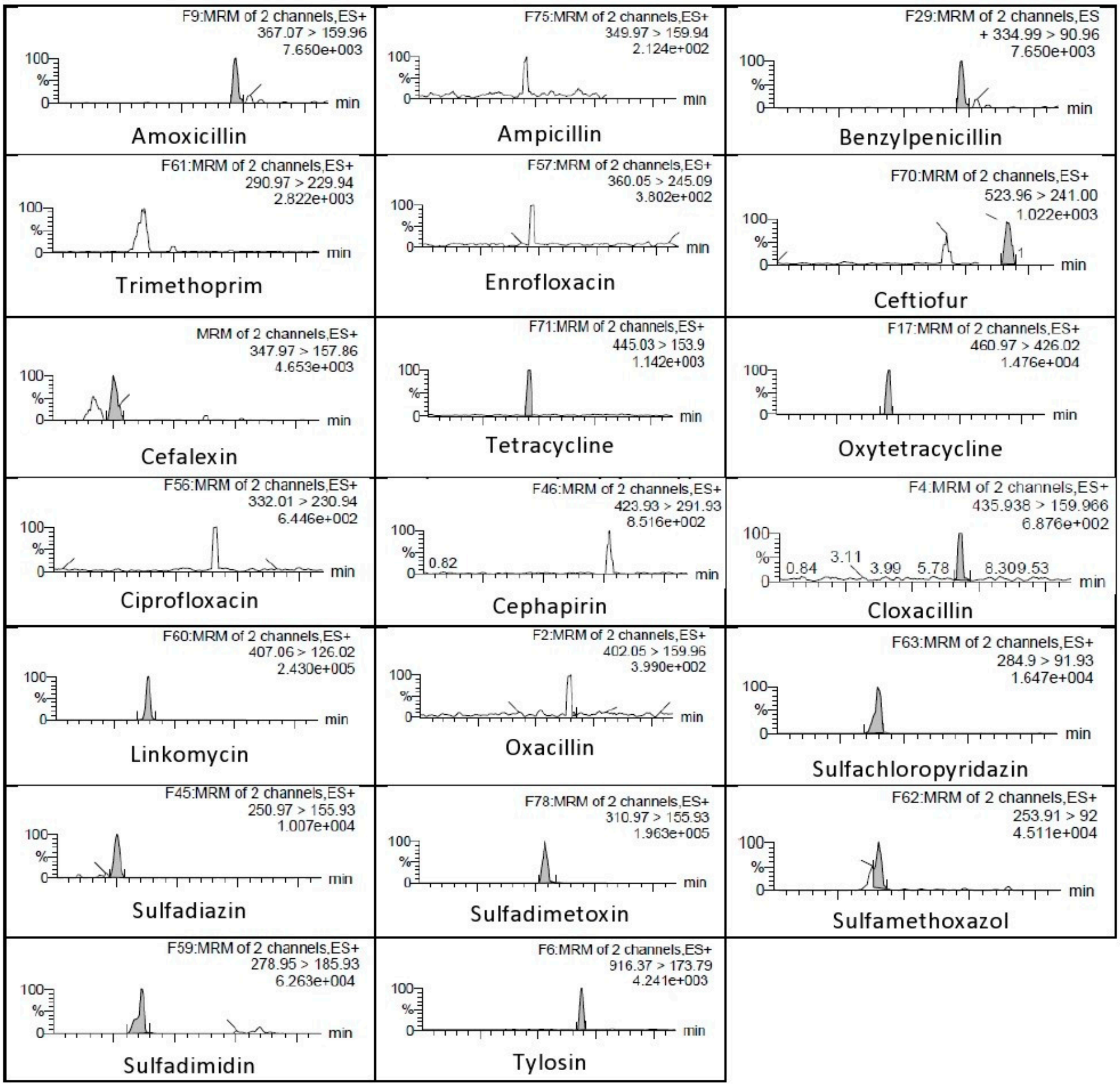

Figure S1. The chromatograms of spiked feces samples

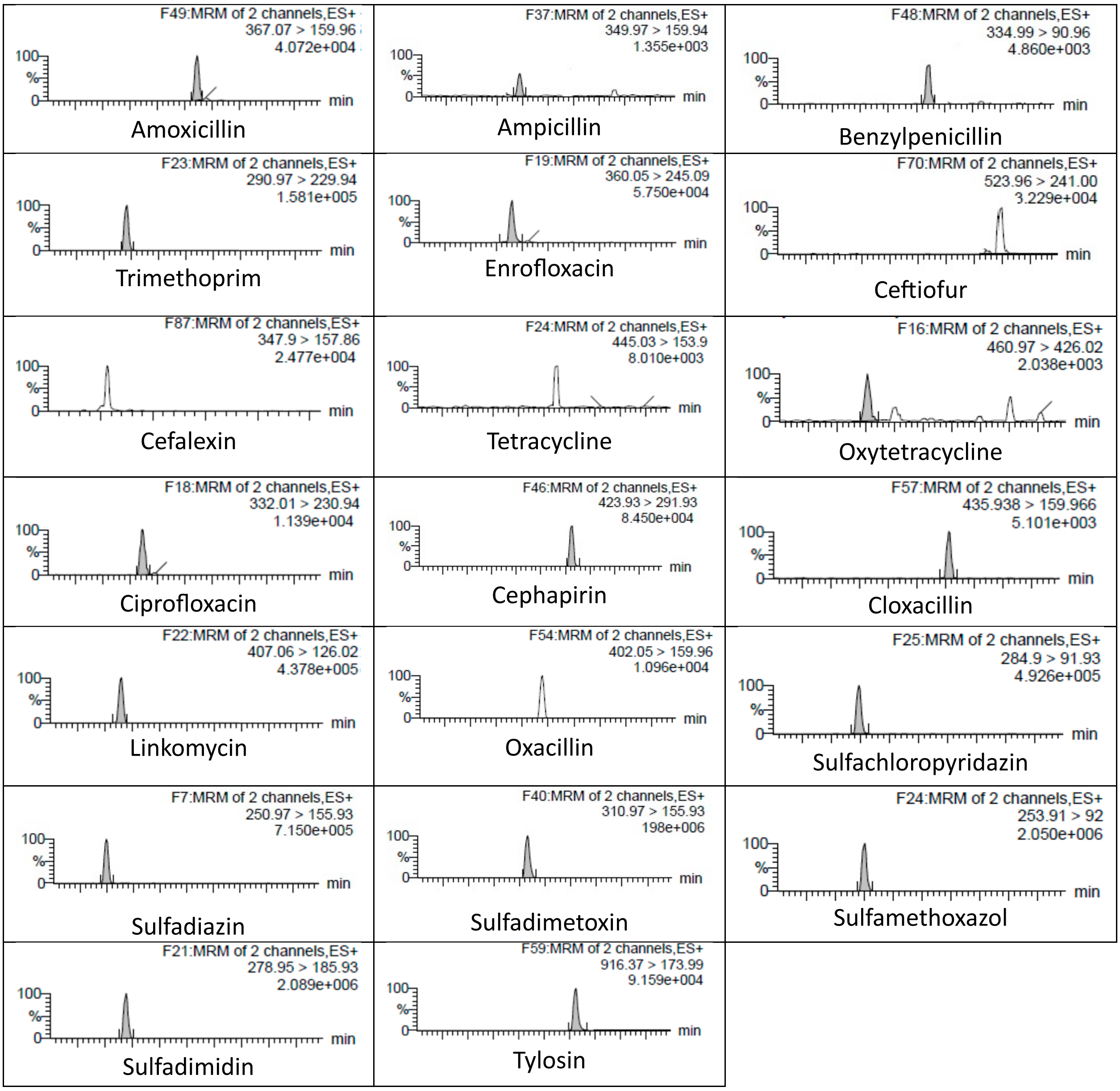

Figure S2. The chromatograms of spiked milk samples
